# Analysis of Chemokine Receptor Trafficking by Site-Specific Biotinylation

**DOI:** 10.1371/journal.pone.0157502

**Published:** 2016-06-16

**Authors:** Marcel Liebick, Christian Schläger, Martin Oppermann

**Affiliations:** Department of Cellular and Molecular Immunology, University of Göttingen, Göttingen, Niedersachsen, Germany; University of Florida, UNITED STATES

## Abstract

Chemokine receptors undergo internalization and desensitization in response to ligand activation. Internalized receptors are either preferentially directed towards recycling pathways (e.g. CCR5) or sorted for proteasomal degradation (e.g. CXCR4). Here we describe a method for the analysis of receptor internalization and recycling based on specific Bir A-mediated biotinylation of an acceptor peptide coupled to the receptor, which allows a more detailed analysis of receptor trafficking compared to classical antibody-based detection methods. Studies on constitutive internalization of the chemokine receptors CXCR4 (12.1% ± 0.99% receptor internalization/h) and CCR5 (13.7% ± 0.68%/h) reveals modulation of these processes by inverse (TAK779; 10.9% ± 0.95%/h) or partial agonists (Met-CCL5; 15.6% ± 0.5%/h). These results suggest an actively driven internalization process. We also demonstrate the advantages of specific biotinylation compared to classical antibody detection during agonist-induced receptor internalization, which may be used for immunofluorescence analysis as well. Site-specific biotinylation may be applicable to studies on trafficking of transmembrane proteins, in general.

## Introduction

Chemokine receptors belong to the family of G protein coupled receptors (GPCR) which form the largest group of signal transducing transmembrane proteins [1,2]. Chemokine receptors and their ligands are expressed on various cell types in different tissues and activate a wide range of downstream effectors due to their nonexclusive agonist repertoire [3]. They are involved in several pathological relevant processes such as metastasis, HIV infection and inflammation [4–8]. Regulation of chemokine receptor expression levels in order to limit chemokine-induced cellular responses is important. The underlying mechanisms are still not well understood.

Several methods have been established to analyze GPCR trafficking. By far the most commonly applied method is direct staining of the receptors or a related tag with fluorochrome-labeled anti-receptor antibodies in combination with flow cytometry [9]. In combination with immunofluorescence this approach can also be used to determine the intracellular receptor distribution [10]. Other less commonly applied methods are based on the quantification of radioligand uptake or on antibody feeding experiments [11,12]. These methods are potentially limited by masking of functionally relevant domains or by unspecific binding which may also facilitate receptor endocytosis [10,13].

These methods are sufficient to detect rapid changes in receptor expression levels but are less well suited for quantification of slower events, e.g. during constitutive internalization. Here the internalization process is obscured by parallel processes such as receptor recycling or translocation of newly synthesized receptors to the plasma membrane. To address this problem we developed a detection method based on specific biotinylation of AP-tagged receptor populations which allows tracking of distinct receptor populations. This approach may be applicable to the study of transmembrane protein trafficking, in more general terms.

Receptor endocytosis is triggered by an agonist-induced conformational rearrangement of the receptor leading to activation of associated G proteins followed by C terminal phosphorylation of receptors via second messenger-dependent protein kinases or GPCR kinases [14–16]. Phosphorylation is crucial for the internalization process whereby alterations in single phosphorylation sites result in critical changes for the internalization process [17,18]. Internalization is mediated by β-arrestin binding which directs the receptor towards clathrin-coated pits [19–21]. Once receptors are internalized and transported to early endosomes they are sorted either for receptor degradation or recycle back to the cell surface [22]. Some chemokine receptors including CCR5 rapidly recycle back to the cell surface to contribute to resensitization while others, such as CXCR4, recycle poorly but are mainly directed into lysosomes for proteosomal degradation [23–26]. These structural similarities and differences in endocytic processing make both receptors interesting candidates to analyze and quantify endocytic trafficking.

We provide quantitative data on the constitutive internalization process of both receptors and its modulation by receptor ant-/agonists. Furthermore, we demonstrate the effect of rapid reinternalization after agonist-induced internalization and its importance for the regulation of the cell surface expression of these receptors.

## Experimental Procedures

### Materials

Cell culture media and additives were from Biochrom, Thermo Fisher Scientific or Invitrogen. Cell culture consumables were from Greiner Bio-One. Chemicals, reagents western blot equipment and further consumables were obtained from Carl Roth, Sigma Aldrich, Sarstedt or Thermo Fisher. Primer and peptides were synthesized by Iba or JPT. Restriction enzymes, ligases and phosphatases were from NEB. DNA purification kits were from Machery & Nagel. Anti-receptor antibodies were from Biolegend and RnD systems. Secondary antibodies and conjugates were from Jackson Immuno Research. Agonists and antagonists were obtained from Merck, Peprotech, Perkin Elmer or Sigma Aldrich.

### Eukaryotic expression systems

Wildtype receptors were modified with an N-terminal AP-tag (GLNDIFEAQKIEWHE) using PCR-based methods. Resulting DNA fragments were ligated in frame into the eukaryotic expression vector system pEF1/Myc-His A and verified using automated Sanger sequencing.

### Cell culture and transfection

Rat basophilic leukemia cells clone 2H3 (RBL 2H3) were transfected by electroporation and selected with 0.6 mg geneticin per ml cell culture medium. Cells were cultivated in RPMI 1640 medium supplemented with 10% heat—inactivated fetal calf serum, 100 μg/ml streptomycin and 100 units/ml penicillin under an atmosphere of 5% CO_2_ at 37°C.

### Generation of anti-AP specific monoclonal antibodies

A peptide corresponding to the amino acid sequence of the AP-tag with an additional C-terminal cysteine residue was synthesized and coupled to maleimide-activated KLH. BALB/c mice were immunized in monthly intervals using 50 μg conjugate with adjuvant. Monoclonal antibodies were generated following standard procedures [27]. Two murine antibodies with specificity against the AP-tag (clone YC8 IgG_2A_/κ; clone EF10 IgG_1_/κ) were selected and further characterized.

### Biotinylation of cell surface receptors

Biotin ligase (BirA) was purified from transformed E. Coli lysates by nickel chelat chromatography and functionally tested by FACS analysis of biotinylated AP-tag expressing cells. AP-tag biotinylation was done in modification of previously described protocols in 10 mM TRIS 30 mM potassiumglutamate buffer system supplemented with 500 mM bicine (pH 8.0), 10 mM ATP, 10 mM magnesium oxaloacetate and 50 μM biotin. Biochemical biotinylation of intact cells was performed using 0.01% biotin X-NHS in phosphate buffer saline (PBS), pH 8 (15 min, room temperature) [28].

### Immunoprecipitation and immunoblotting

Biotinylated or non-biotinylated RBL cells were solubilized in detergent buffer supplemented with 50 mM Tris-HCl, pH 8.0, 150 mM NaCl, 5 mM EDTA, 1% Triton X-100, 0.05% SDS, 10 mM NaF, 10 mM Na_2_HPO_4_ and 1:500 phenylmethanesulfonyl fluoride (PMSF) (10 min, on ice). Immunoprecipitation was done using streptavidin-sepharose (StAv-sepharose) (90 min, 4°C). Samples were extensively washed with detergent buffer, dissolved in SDS sample buffer containing 62.5 mM Tris-HCl, 2% SDS, 10% glycerol, 0.05% bromophenol blue and 10% β-mercaptoethanol and separated using electrophoresis on a 10% SDS polyacrylamide gel matrix. Immunoblotting was done using horseradish peroxidase (HRP) coupled anti-CCR5 R22/7 antibody and StAv-HRP in TRIS-buffered saline (TBS) supplemented with 0.1% Tween-20 and 1% bovine serum albumin (BSA) (60 min, rt). Proteins were detected by chemiluminescence.

### Internalization/recycling and flow cytometry

Receptor expression levels were determined using anti-receptor or anti-AP antibodies by flow cytometry.

For the study of constitutive receptor internalization cells were resuspended in potassium glutamate buffer containing 3 μM BirA and incubated at rt (30 min). Cells were washed with binding medium (BM; RPMI 1640, 0.2% BSA, 10 mM HEPES, pH 7.4) in which they were incubated up to 4 hours at 37°C. For the analysis of agonist-induced internalization biotinylated cells were treated accordingly and 125 nM agonist was added (30 min, 37°C). Unbound ligand was removed by acid wash with EM medium (RPMI 1640, 0.2. % BSA, 10 mM MES, pH 2.5) at 4°C. Receptor recycling was measured with cells incubated in agonist-free BM medium in the presence or absence of antagonist (AMD3100 30 μM; TAK779 3 μM) (30 min, 37°C). Biotinylated receptors were stained with phycoerythrin-labeled streptavidin (StAv-PE) and detected by flow cytometry. Classical antibody-based detection was done accordingly by staining with anti-AP antibodies. Receptor internalization and recycling was calculated as percentage of expressed receptors on the cell surface at time 0 min.

### Immunofluorescence

RBL cells were grown on glass coverslips in 24-well plates (overnight, 37°C). Cells were biotinylated, stimulated and fixed with 3% paraformaldehyde in PBS (20 min, 37°C). Reactive aldehyde groups were saturated with 50 mM NH_4_Cl in PBS (30 min, 37°C). Cells were permeabilized with PBS supplemented with 0.1% saponin and 0.2% gelatin (15 min, 37°C). Staining was done using anti-AP antibody (10 μg/ml) and StAv-Alexa647 (2 μg/ml) (60 min, on ice). After washing with PBS-saponin Alexa488 labeled goat anti mouse Ig was used as secondary antibody (60 min, on ice). After extensive washing, cells were mounted with Mowiol supplemented with 0.1% p-phenylenediamine. Cells were evaluated using a confocal laser microscope.

## Results

### Expression of N-terminally AP-tagged chemokine receptors and generation of an AP-tag specific antibody

**Fig 1:**
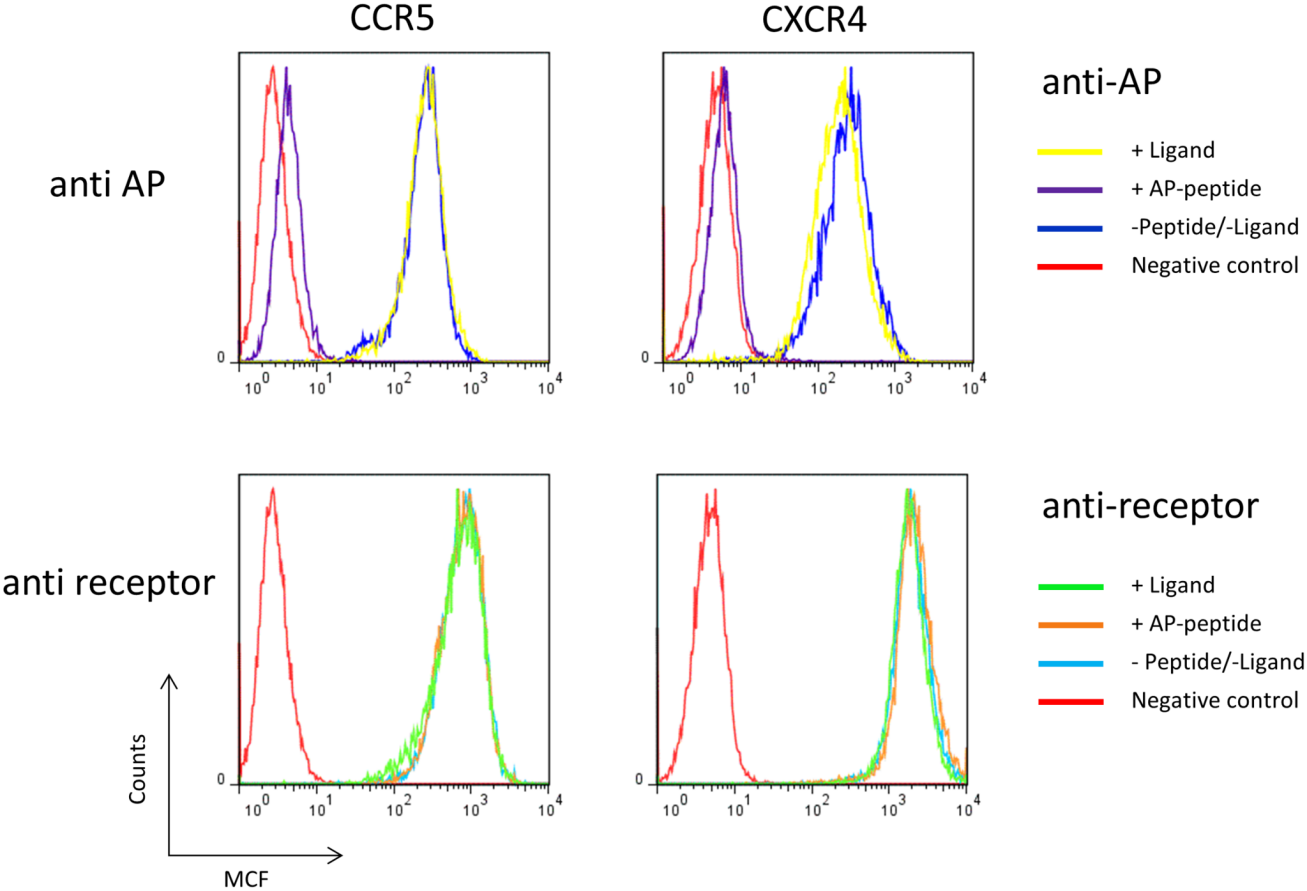
Binding of anti-AP and anti-receptor antibodies to RBL-CXCR4/CCR5-cells. RBL-CXR4-AP (right) or RBL-CCR5-AP (left) cells were stained (60’/4°C) with 10 μg anti-AP (upper panels) or 1.5 μg PE-labeled anti-receptor antibody (anti CXCR4 12G5, anti-CCR5 T21/8) (lower panels) after preabsorption of antibodies with 15 μg AP-peptide (30 ‘/rt) or pretreatment of receptor-expressing cells with 50 nM ligand (CCL5/CXCL12) (10’/4°C). Receptor-bound antibodies were detected with FITC-labeled anti-mouse IgG antibodies. RBL-2H3 cells served as negative control. Representative diagrams show the mean channel of fluorescence (MCF) in correlation to detected events.

To analyze internalization and recycling of distinct receptor populations RBL-cells were stably transfected via electroporation with AP-tagged versions of CXCR4 and CCR5. Comparable expression levels were confirmed by quantitative FACS analysis, functional integrity was shown by ligand-induced N-acetyl-β-D-glucosaminidase (NAGA) release (data not shown). To specifically detect the AP-tag monoclonal antibodies were generated according to standard techniques. AP-tag specificity was confirmed by ELISA and flow cytometry. Detection of AP-tagged receptors was fully abrogated after pre-absorption of anti-AP antibodies with an excess of AP-peptide (Fig 1). Pretreatment of anti-receptor antibodies with AP-peptide did not result in a decreased binding capacity. Ligand stimulation did not affect anti-AP or anti-receptor antibody binding thus specific interaction between anti-AP antibody and AP-tag does not interfere with ligand-binding.

### AP-tag specific biotinylation mediated by BirA

**Fig 2:**
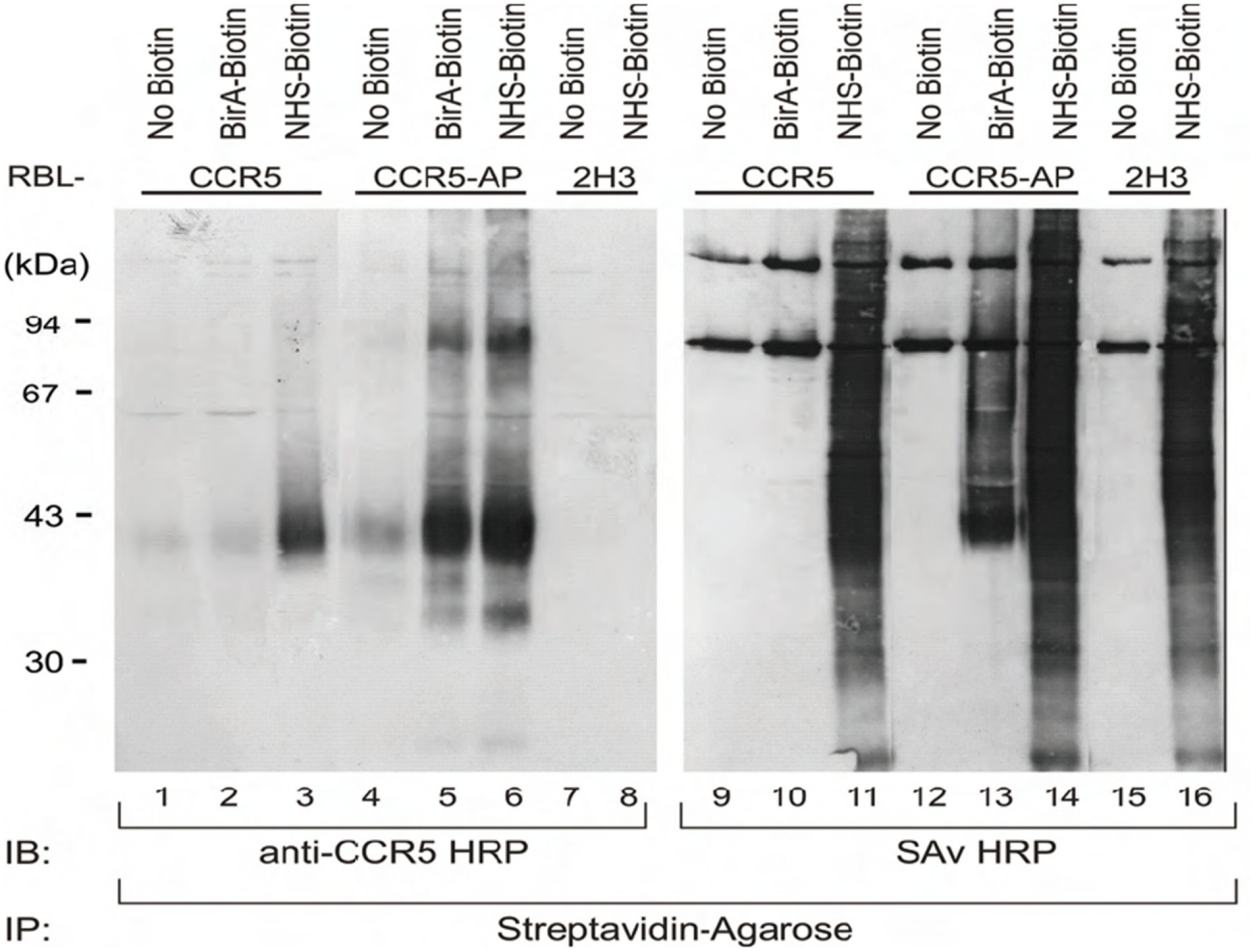
BirA-mediated biotinylation of AP-tagged CCR5 on RBL cells. RBL cells (2H3) expressing CCR5, CCR5-AP or no receptor (2H3) were either biotinylated with BirA (BirA-Biotin) or biotin-X-NHS (NHS-Biotin; 15’/rt). Cells were lysed (10’) and immunoprecipitated via streptavidin agarose (90’/4°C). Samples were probed by immunoblotting with anti-CCR5 (T21/8; left) or streptavidin peroxidase conjugates (right).

In contrast to conventional primary amine biotinylation BirA catalyzes the biotinylation of one specific lysine residue within the AP-tag sequence [29]. In order to test the ability of BirA to specifically biotinylate AP-tagged receptors we immunoprecipitated enzymatically and biochemically biotinylated CCR5- and CCR5-AP receptors in parallel (Fig 2). Precipitates detected with anti-CCR5 antibodies showed a positive chemical biotinylation of CCR5 and CCR5-AP expressing cells independent of the presence of AP-tag (lane 3 and 6). However, enzymatic biotinylation allowed immunoprecipitation and detection only of AP-tagged CCR5 receptors, this showing the efficiency and specificity of the biotinylation process (lane 2 and 5). When detected with streptavidine only enzymatically biotinylated CCR5-AP receptors showed a distinct band (lanes 10 and 13) whereas biotinylation with NHS-biotin resulted in unspecific biotinylation of cell surface proteins regardless of the presence or absence of the AP-tag, underlining the specificity of the biotinylation reaction (lanes 11; 14 and 16). The distinct band at 43 kDa was indeed biotinylated CCR5 receptor as verified by immunodetection with anti-CCR5 antibodies (lanes 5 and 13).

### Constitutive internalization of CXCR4 and CCR5 and modulation by receptor ant-/ agonists

Membrane-expressed receptors undergo a constant cycle of ligand-independent, constitutive internalization and re-expression at the cell surface. In order to describe this process in quantitative terms untreated cells were enzymatically biotinylated and stained either with anti-AP antibodies or streptavidine.

**Fig 3:**
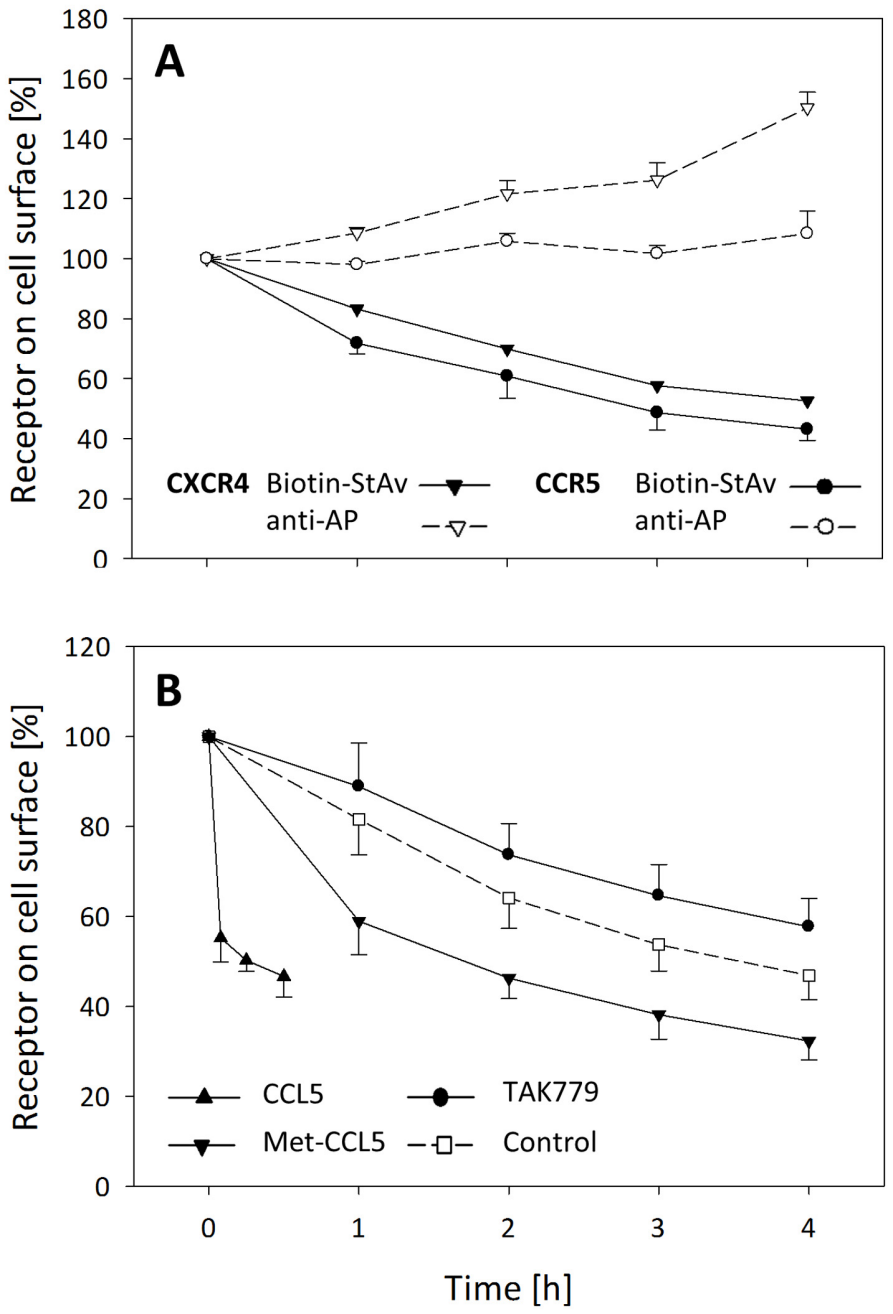
Constitutive receptor internalization and its modulation by receptor ant-/agonists. A: RBL cells stably expressing CXCR4-AP or CCR5-AP were enzymatically biotinylated and incubated in BM medium for up to four hours (37°C). Cells were stained with anti-AP (dashed lines) or streptavidin (straight lines) and analyzed by flow cytometry. B: CCR5-AP cells were either treated with (filled symbols) or without (open symbols) ant-/agonists (CCL5 0.05 μM; Met-CCL5 0.15 μM; TAK779 3 μM) and stained in accordance to A. All results represent the mean value +/- s.d. of at least three independent experiments.

Cells stained with classical antibody-based detection (anti—AP) showed no significant alterations in receptor expression levels (Fig 3A; dashed lines). However, using the biotin detection system we observed a constant decrease of receptors expressed at the cell surface (Fig 3A; straight lines). In the absence of receptor ligand CXCR4 showed a constitutive internalization rate of 12.1%/h ± 0.99%. With 13.7%/h ± 0.68% the internalization rate of CCR5 was slightly higher compared to CXCR4.

Several CCR5 ant-/agonists have been reported to interact with the receptor and thereby modulate receptor internalization. To analyze the consequences of antagonist binding for the internalization process cells were treated as previously described and incubated in the presence of CCR5- ant-/agonists.

The inverse CCR5 agonist TAK779 inhibits basal activity of the receptor. Receptors incubated with TAK779 were significantly slower internalized (10.9%/h ± 0.95%) compared to untreated cells (13.4%/h ± 0.88%) (Fig 3B; circle and square). Treatment with partial or full agonists of CCR5 (Met-CCL5 and CCL5), which both induce receptor activation activity over basal levels resulted in an enhanced internalization of either 15.6%/h ± 0.5% for Met-CCL5 or 9.3%/min ± 1.0% for the natural ligand CCL5 (Fig 3B; triangle down/up).

Taken together, specific biotin labeling of chemokine receptors at the cell surface revealed their internalization at a constant low rate of 12.1–13.7% per hour in the absence of external stimuli. Internalization is significantly retarded (10.9%) in the presence of an inverse agonist. These minor changes in receptor expression are obscured by receptor recycling and may not be monitored by classical antibody-mediated detection methods.

### Quantification of ligand-activated receptor internalization and recycling in the presence or absence of receptor antagonists

**Fig 4:**
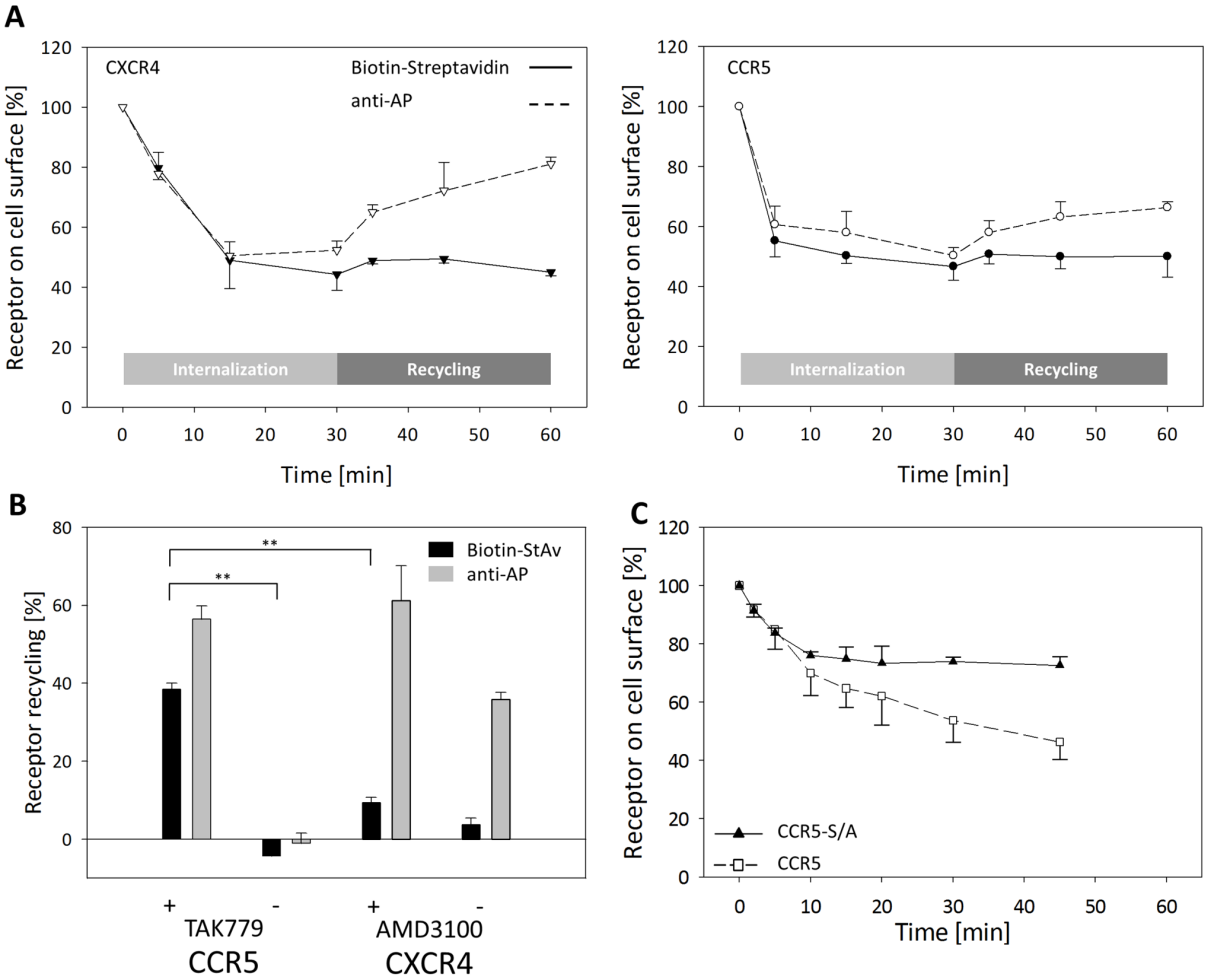
Ligand-induced internalization and recycling in presence or absence of receptor antagonists. A: RBL cells stably expressing CXCR4- (left) or CCR5-AP (right) were enzymatically biotinylated and incubated in BM medium containing 125 nM ligand (30’/37°C). Recycling was triggered by acid wash to remove the ligand and transfer into ligand-free medium (30’/37°C). Cells were stained with anti-AP (dashed line) or streptavidin (straight line) and analyzed by flow cytometry. Each curve shows the mean percentage (+/- s.d.) of receptors expressed on the cell surface normalized to the MCF value of untreated cells. B: Cells were treated and stained accordingly to panel A (grey bars anti-AP, black bars streptavidin). During the recycling phase the corresponding antagonist was added (TAK779 3 μM; AMD3100 30 μM). C: RBL cells expressing CCR5-AP S/A (filled triangle up) or CCR5-AP WT (open square) were enzymatically biotinylated. Internalization was triggered as described in panel A. Staining was done with streptavidin-PE. Receptor recycling was calculated as percentage of the difference between cell surface expression of the receptor at time points 0 and 30 minutes Results represent mean +/- s.d. of at least three independent experiments. n.s., not significant; **, p < 0.001).

To compare classical antibody-based and the newly established biotin-based detection assay during agonist driven receptor internalization we stained enzymatically biotinylated CXCR4-/CCR5-AP expressing cells either with anti-AP or streptavidin. Upon agonist stimulation both receptors were rapidly and equally well internalized with both detection methods (Fig 4A). However, after removal of the agonist in both cell lines a significant recycling of anti-AP stained cell in contrast to the non-recycling biotinylated receptors was observed. Previous studies showed a different recycling behavior of CXCR4 and CCR5 where CCR5 preferentially recycles back to the cell surface whereas CXCR4 stays within the cytoplasm for proteasomal degradation [25,30]. These results are in contrast to our finding of lack of receptor recycling. To determine whether recycled receptors rapidly re-internalize after receptor recycling to the cell surface we used the receptor antagonists TAK779 and AMD3100 to lock down recycled receptors at the cell surface. Biotinylated CCR5 receptors treated with TAK779 showed a significantly enhanced receptor recycling compared to untreated cells (Fig 4B left, black bars). This result was confirmed by anti-AP staining (Fig 4B left, grey bars). The same effect was observed for CXCR4 (Fig 4B right), although to a lower degree. Comparison of the recycling rates of biotinylated CXCR4 and CCR5 showed that while CCR5 recycled back to the cell surface CXCR4 was retained within the cell (Fig 4B, black bars). These data suggest that CCR5 receptors indeed recycle but also tend to rapidly re-internalize which cannot be shown by classical antibody-based detection.

Since phosphorylation of the receptor C terminus was previously shown to be critical for receptor internalization we generated a phosphorylation-deficient mutant in which all potential serine phosphorylation sites were changed to alanine (S/A). Internalization of biotinylated receptors was significantly impaired compared to the wildtype receptor (Fig 4C). During prolonged agonist stimulation this effect was even more evident, with 20% less receptor internalization of the S/A mutant compared to wildtype. This finding confirms previous studies showing that truncation or Ser/Ala mutation of the receptor C-terminus results in impaired endocytic receptor trafficking.

### Intracellular distribution of internalized and pre-stored receptors determined by immunofluorescence

BirA-mediated labelling of distinct receptor populations at the cell surface may be used to follow their intracellular fate during agonist-induced internalization and recycling using immunofluorescence microscopy.

**Fig 5:**
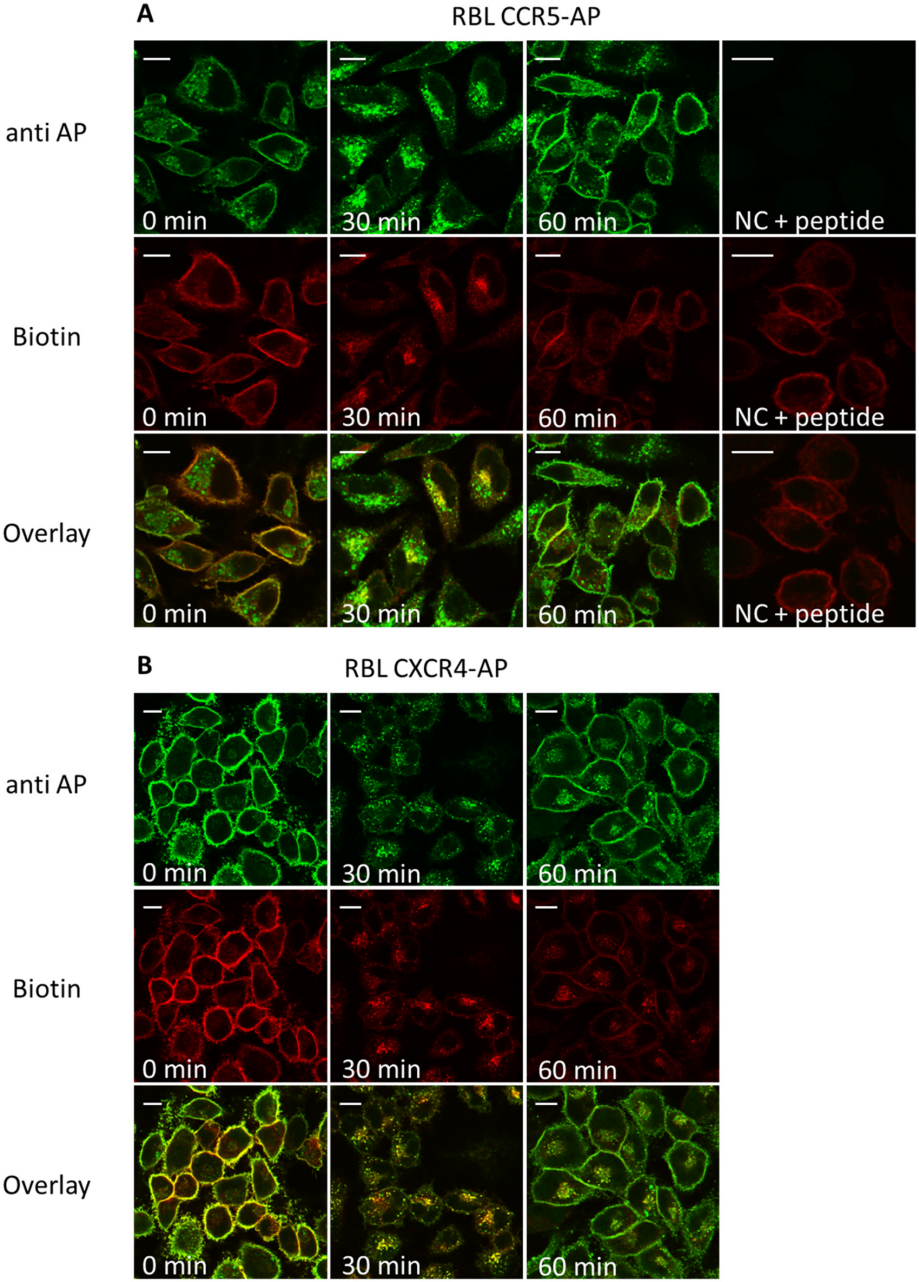
Double immunoflourescence of CXCR4-/CCR5-expressing cells during ligand-induced internalization (30’) and recycling (60’). Prior to stimulation and staining CXCR4-AP (bottom) and CCR5-AP (top) expressing RBL cells were seeded on glass cover slips. Cells were enzymatically biotinylated and stimulated with the corresponding ligand (125 nM CCL5, CXCL12; 30’/37°C). Recycling was induced by acid wash and transfer into ligand-free medium containing receptor antagonists (30 μM AMD 3100; 3 μM TAK779). Cells were fixed with 3% PFA (15’/37°C) and permeabilized with 0.1% saponin (15’/37°C). 10 μg/ml anti-AP antibody (anti AP; with or without preincubation with 2mg /ml of the AP-peptide) and 2 μg/ml streptavidin-Alexa647 (Biotin) was used for staining (60’/on ice/dark). Samples were fixed with mounting medium and analyzed by confocal laser scanning microscopy. Scale bar 10 μM.

In the absence of agonist a clear and distinct staining at the cell surface was observed with staining of additional internal vesicular structures using anti-AP antibodies (Fig 5). Upon agonist stimulation, receptors started to redistribute into distinct punctate vesicles and this correlated with a significant reduction of cell surface expression. However, internalized CCR5 receptors did not fully co-localize with the pre-stored receptor subpopulation but instead were found in a distinct niche in the perinuclear area (Fig 5, top, 30 min). This shows the potential of the biotin system to follow the fate of internalized receptors, whereas classical antibody-based detection shows no difference between internalized and pre-stored receptors. The internal distribution of vesicular structures differed significantly between CCR5 which was localized in close proximity to the perinuclear area and CXCR4 which displayed a more diffuse distribution. After removal of the agonist membrane expression of both receptors was restored. However, biotinylated CXCR4 receptors were preferably retained in the cytoplasm compared to CCR5 receptors. Taken together, CXCR4 and CCR5 differ significantly in the distribution of internalized receptors which may also correlate with their different recycling behavior as reported before.

## Discussion

Chemokine-chemokine receptors axes control migration and positioning of immune cells in physiological (homeostasis) and pathological conditions e.g., inflammation [31,32]. Chemokine receptors and their ligands are associated with several diseases and play also a crucial role in tumor progression and metastasis [33–36]. Chemokine receptor trafficking is still a subject of intensive research [26]. Several methods have been established to quantify intracellular GPCR trafficking, while still the most commonly used method is based on classical antibody staining [9,37]. Antibody-based methods are sufficient to detect rapid changes in receptor expression levels but are less applicable to the study of less pronounced changes in receptor expression since they are potentially affected by cycling and newly synthesized receptors. These limitations were addressed by dye-conjugated antibodies which target extracellular epitopes and selectively label subpopulations of expressed receptors in a process known as “antibody feeding” [11,38]. Both methods are also suitable for the intracellular detection of internalized receptors by immunofluorescence microscopy [10,39]. However, a clear differentiation between internalized receptors and those which are pre-stored in intracellular compartments is still a technical challenge [40]. Other methods were devised and optimized for high-throughput-screening (HTS) of receptor internalization including ELISA, enzyme-based assays (lactamase, galactosidase complementation and luciferase), fluorogen activating protein (FAP) and fluorescence resonance energy transfer (FRET) [41–46]. Most recently, a technique based on diffusion-enhanced resonance energy transfer (DERET) was applied, which is easy to implement and combines the advantages of single labeling accompanied with a high signal to noise ratio [47]. However, DERET is not suited for the analysis of endogenous expressed receptors.

We developed an alternative method for the quantitative analysis of receptor trafficking which is based on the specific biotinylation of receptors at the cell surface with biotin ligase (Bir A) (28,48). BirA catalyzes the covalent biotinylation of a 15 aa peptide (AP-tag) sequence on cell surface proteins [29,48]. Various proteins have been biotinylated via the AP-tag for different purposes, including in vitro biotinylation [28,49–53]. We refined this approach to quantify endocytic trafficking of CXCR4/CCR5 without having to consider translocation of newly synthesized receptors towards the plasma membrane. To simultaneously detect all receptors expressed within a cell we generated an anti-AP specific monoclonal antibody, which binds to the AP-tag even in the presence of prior ligand or streptavidin binding. The system may be also used for double immunofluorescence analysis to discriminate between pre-stored receptors and actively internalized (biotinylated) receptors, which is not easily achieved with previously described methods. In contrast to similar techniques based on the modification of acyl carrier protein (ACP) by phosphopantetheine transferase (PPTase) our method does not allow direct labeling with dye-conjugated biotin. This further reduces the potential to quantify highly dynamic processes during receptor trafficking [54]. In general, enzyme-based detection methods require certain buffer conditions which can differ significantly from normal cell culture conditions. Furthermore enzymatic reactions are susceptible to interference and therefore a constant efficiency level is not guaranteed.

In this study we used the BirA-based biotinylation system to determine constitutive internalization of CXCR4 and CCR5. Internalization rates for both receptors were at a comparable level (CXCR4 12.1%/h ± 0.99%, CCR5 13.7%/h ± 0.68%), which suggests a slow but constant flow of receptors into endocytic compartments.

Currently it is still a matter of debate whether constitutive receptor internalization is merely a consequence of normal turnover of the plasma membrane in a passive manner or whether it is attributed to an equilibrium between active and inactive forms of the receptor where the subpopulation of active receptors undergoes constitutive internalization [55–57]. In recent studies several ant-/agonists showed their potential to directly influence receptor internalization [58,59]. To analyze the effect of antagonist binding on receptor internalization and to further address the question of active vs. passive internalization we treated CCR5 expressing cells with the inverse agonist TAK779 and the partial agonist Met-CCL5 [59,60]. Compared to untreated cells (13.9%/h) Met-CCL5 treatment induced an enhanced internalization of CCR5 (15.6%/h) whereas TAK779 treated receptors were internalized at significantly lower levels (10.9%/h). In recent studies it was demonstrated that the functional versatility of GPCRs is highly dependent on structural plasticity, which is also the reason for receptors to provoke a basal level of activity in the absence of an endogenous agonist [61]. Thereby basal activity is accompanied with C-terminal phosphorylation and β-arrestin-binding [62–65]. Binding of ant-/agonists interferes with general plasticity of the receptor by stabilizing a discrete receptor conformation which is either susceptible to C-terminal phosphorylation or not [61,66–69]. Our results show it is very likely that changes in receptor conformation are indeed the main reason for constitutive receptor internalization. Binding of Met-CCL5 or TAK779 stabilizes the receptor in confirmations favoring C-terminal phosphorylation and consecutive β-arrestin binding or not. Whereas Met-CCL5 binding only partially supports C-terminal phosphorylation the full agonist CCL5 appears to fully stabilize an active receptor conformation which results in a robust internalization at an initial rate of 9.3%/min ± 1.0%.

In contrast to constitutive internalization which came into focus recently, agonist-induced internalization and different processing of internalized CXCR4 and CCR5 receptors has been studied for over two decades [23,26,30,70,71]. In our study receptor recycling of CXCR4 and CCR5 correlated with the biotinylation status of the receptor (Fig 4A) which is noteworthy since previous studies were mostly done using non-biotinylated receptors and classical antibody detection [23,72]. In previous studies it was reported that in contrast to other GPCRs vesicular acidification is not mandatory for CCR5 to initiate receptor recycling [73–76]. Instead, ligand-occupied and phosphorylated CCR5 receptors are presumably re-expressed at the cell surface to transit the endocytic machinery multiple times without repeated stimulation [30,76,77]. To exclude the effect of rapid receptor reinternalization we treated cells with the inverse agonist TAK779 to actively displace bound agonist from recycled receptors and lock them at the cell surface [76,78]. TAK779-treated CCR5-expressing cells showed increased recycling compared to untreated cells (Fig 4B, left) indicating that rapid reinternalization indeed interferes with the quantification of recycled receptors. Similar results were obtained after AM3100 treatment of CXCR4-expressing cells (Fig 4B, right). Lower recycling rates of biotinylated and AMD3100-treated CXCR4 cells compared to CCR5 cells may result of partial agonism of AMD3100 or subsequent ubiquitination and degradation of internalized CXCR4 receptors [25,63,71]. Differences in the recycling characteristics of biotinylated and non-biotinylated receptors may refer to translocation of stored receptors from the trans-Golgi network towards the cell surface [79]. However, this difference still needs to be further addressed since a majority of earlier studies were done using direct staining of non-biotinylated receptors.

Over the years the different intracellular distribution of internalized CXCR4 and CCR5 receptors was studied in detail [59,80,81]. Although a perinuclear localization of internalized CCR5 receptors had been shown different subcompartments for the site of receptor accumulation were taken into consideration including early endosomes, the endosomal recycling compartment and the golgi apparatus [30,58,59,76,79,82–84]. In our studies we combined detection of biotinylated and non-biotinylated receptors during immunofluorescence analysis to allow a distinct differentiation between newly internalized and cycling receptors in agonist stimulated cells (Fig 5, 30 min). CCR5-expressing cells showed a delineation of cycling and biotinylated receptors which supports the hypothesis of accumulation of internalized receptors in the trans golgi region in close proximity to cycling and newly synthesized receptors [79,85]. However, it cannot entirely ruled out that receptor accumulation already occurs in the endosomal recycling compartment because of a fluent transition between both compartments [30,79,84,85]. In agonist-treated CXCR4-expressing cells no distinct accumulation of internalized receptors was observed which indicates proteasomal degradation of CXCR4 in diffusely distributed lysosomes [22,23,25,85]. However, a small amount of internalized CXCR4 receptors can recycle back to the cell surface [71,86]. According to our data this is not accompanied by detectable receptor accumulation in distinct compartments.

In summary, we provide additional evidence that constitutive internalization is indeed an active process which is dependent on the structural plasticity of the receptor. Therefore, binding of receptor ant-/agonists can positively or negatively influence this active process by stabilizing distinct receptor conformations. Recycling studies of biotinylated receptors supported the hypothesis that CCR5 receptors transit the endocytic machinery multiple times whereas CXCR4 receptors were predominantly retained in the cytoplasm. Immunofluorescence analysis of biotinylated and non-biotinylated receptors showed that internalized CCR5 receptors accumulate in close proximity to cycling receptors whereas CXCR 4 receptors showed no such accumulation.

Overall this biotin-based detection system may be generally applicable to the analysis of transmembrane protein trafficking.
